# A silk-based self-adaptive flexible opto-electro neural probe

**DOI:** 10.1038/s41378-022-00461-4

**Published:** 2022-11-08

**Authors:** Yu Zhou, Chi Gu, Jizhi Liang, Bohan Zhang, Huiran Yang, Zhitao Zhou, Meng Li, Liuyang Sun, Tiger H. Tao, Xiaoling Wei

**Affiliations:** 1 State Key Laboratory of Transducer Technology, Shanghai Institute of Microsystem and Information Technology, Chinese Academy of Sciences, 200050 Shanghai, China; 2 School of Graduate Study, University of Chinese Academy of Sciences, 100049 Beijing, China; 3 School of Physical Science and Technology, ShanghaiTech University, 200031 Shanghai, China; 4 2020 X-Lab, Shanghai Institute of Microsystem and Information Technology, Chinese Academy of Sciences, 200050 Shanghai, China; 5 Center of Materials Science and Optoelectronics Engineering, University of Chinese Academy of Sciences, 100049 Beijing, China; 6 Center for Excellence in Brain Science and Intelligence Technology, Chinese Academy of Sciences, 200031 Shanghai, China; 7 Neuroxess Co., Ltd. (Jiangxi), 330029 Nanchang, Jiangxi China; 8 Guangdong Institute of Intelligence Science and Technology, Hengqin, 519031 Zhuhai, Guangdong China; 9 Tianqiao and Chrissy Chen Institute for Translational Research, Shanghai, China

**Keywords:** Electrical and electronic engineering, Biosensors

## Abstract

The combination of optogenetics and electrophysiological recording enables high-precision bidirectional interactions between neural interfaces and neural circuits, which provides a promising approach for the study of progressive neurophysiological phenomena. Opto-electrophysiological neural probes with sufficient flexibility and biocompatibility are desirable to match the low mechanical stiffness of brain tissue for chronic reliable performance. However, lack of rigidity poses challenges for the accurate implantation of flexible neural probes with less invasiveness. Herein, we report a hybrid probe (Silk-Optrode) consisting of a silk protein optical fiber and multiple flexible microelectrode arrays. The Silk-Optrode can be accurately inserted into the brain and perform synchronized optogenetic stimulation and multichannel recording in freely behaving animals. Silk plays an important role due to its high transparency, excellent biocompatibility, and mechanical controllability. Through the hydration of the silk optical fiber, the Silk-Optrode probe enables itself to actively adapt to the environment after implantation and reduce its own mechanical stiffness to implant into the brain with high fidelity while maintaining mechanical compliance with the surrounding tissue. The probes with 128 recording channels can detect high-yield well-isolated single units while performing intracranial light stimulation with low optical losses, surpassing previous work of a similar type. Two months of post-surgery results suggested that as-reported Silk-Optrode probes exhibit better implant-neural interfaces with less immunoreactive glial responses and tissue lesions.

**Figure:**
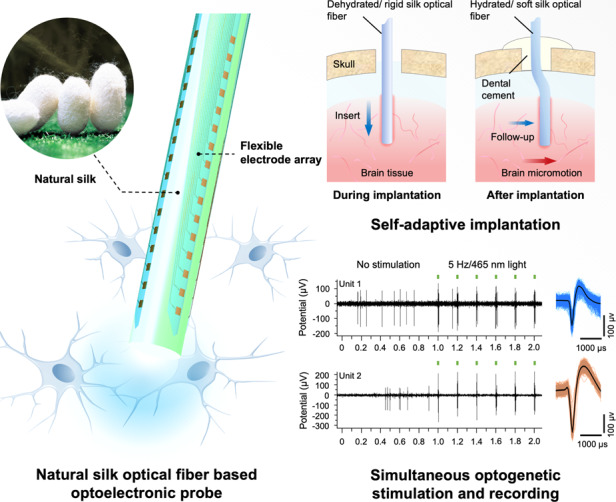
A silk optical fiber-based Silk-Optrode probe consisting of a natural silk optical fiber and a flexible micro/nano electrode array is reported. The multifunctional soft probe can modify its own Young’s modulus through hydration to achieve accurate implantation into the brain. The low optical loss and single-unit recording abilities allow simultaneous optogenetic stimulation and multichannel readout, which expands the applications in the operation and parsing of neural circuits in behavioral animals.

A silk optical fiber-based Silk-Optrode probe consisting of a natural silk optical fiber and a flexible micro/nano electrode array is reported. The multifunctional soft probe can modify its own Young’s modulus through hydration to achieve accurate implantation into the brain. The low optical loss and single-unit recording abilities allow simultaneous optogenetic stimulation and multichannel readout, which expands the applications in the operation and parsing of neural circuits in behavioral animals.

## Introduction

Highly precise optogenetic and electrophysiological studies provide an approach to correlate the neural activity of specific cells to observed behaviors, which is important for the explorations of neural circuits in the brain and the pathologies of the nervous system^1–3^. Embedded optical fibers^4,5^ and electrode probes^6–9^ are the most common invasive devices in the studies of these areas. Previous studies have shown that foreign body reactions due to chronic tissue damage lead to gradual encapsulation of the implanted probes, resulting in dysfunction of the devices^10^. Over the past two decades, researchers have experimented with advanced materials to fabricate invasive electrodes^10–14^ and optical fiber^15,16^ devices that are mechanically compatible with soft brain tissue, thereby reducing the relative micromotion and damage between the device interface and brain tissue^17^. Extensive efforts have been made in this area; however, challenges have yet to be overcome for practical applications. First, the assembly of the optical fiber and electrode(s) generally results in a significant increase in the volume of the implant^5^, which produces additional tissue damage. Second, an alternative strategy is accommodating recording and waveguide channels in fibers through a thermally drawn process^18,19^. However, this method has difficulty breaking the limit of the number of recorded sites (usually less than 30)^20^. Third, existing optical waveguide materials, such as polycarbonate (e.g., 2–2.4 GPa) and quartz (e.g., 77–85 GPa), have a Young’s modulus far greater than that of brain tissue (e.g., 1–10 kPa)^21^. Probes made of softer biocompatible materials, on the other hand, are mechanically incapable of embedding themselves into brain tissue^22^. Moreover, the lack of rigidity poses challenges in the implantation into living brains. A rigid shuttle device is usually required for the implantation of a flexible probe. However, the insertion and retraction of the shuttle device inevitably cause extra damage to the brain tissue.

Natural silk has remarkable application prospects in biomedical^23,24^ and bio-optical devices^25,26^ due to its excellent physical and biomedical properties, such as transparency, mechanical strength and crucial biocompatibility^27^. Here, we present an optoelectronic probe, Silk-Optrode, with natural silk as an optical waveguide material, which can simultaneously achieve high-precision intracranial light stimulation and electrophysiological recording. Through the attachment of the flexible electrode array to the surface of the silk optical fiber, 128 recording channels can be integrated on a single probe. Meanwhile, the diameter of the probe does not notably increase. Compared with polylactic acid (PLA) and other polymer materials^28,29^ as previously reported for making implantable flexible optical fibers, silk has the advantage of being adjustable under different hydration conditions. The Silk-Optrode can actively transform from a hard brittle state to a flexible and high elongation state after implantation (bending stiffness decreased by ~11 times), thus simultaneously achieving the simplest implant operation and causing less chronic tissue damage. Low optical transmission loss makes silk-based optical fibers suitable for optogenetic studies as waveguides. In these studies, Silk-Optrode achieved the effective regulation of neural activity and behavior while recording more than 50 high-quality isolated unit activities within one experiment. We envision that this technology will provide new opportunities for the combination of multifunctional biomaterial devices with neurological disease research.

## Results

### Fabrication and characterization of flexible silk optical fibers

**Fig. 1:**
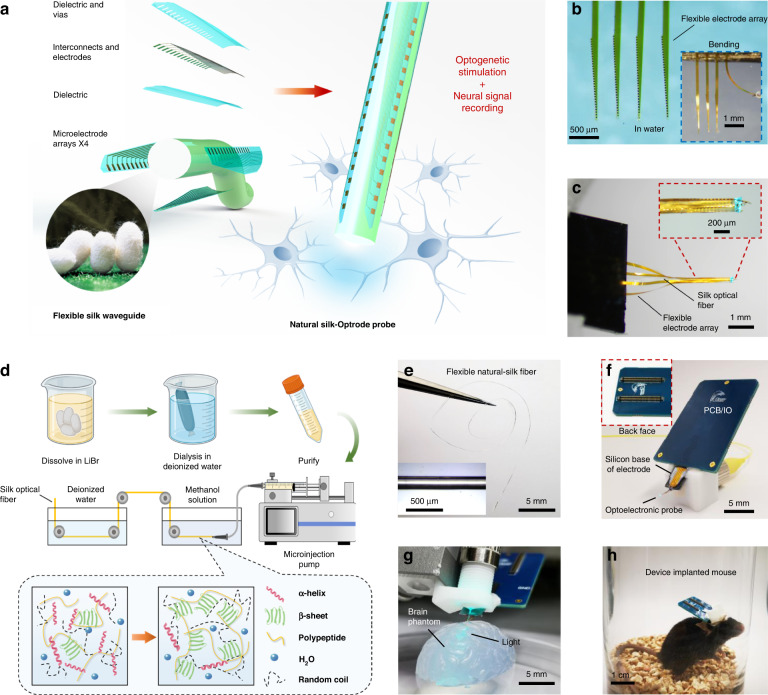
Silk optical fiber-based Silk-Optrode probe, with multichannel electrode array and natural silk waveguide. **a** A schematic to demonstrate the concept of a silk optical fiber-based Silk-Optrode probe and the multilayer structure of the device. **b** Microscopic image of the released ultrathin flexible electrode array. **c** Microscopic photograph of the Silk-Optrode probe connected to the laser light source. **d** Production process of the natural silk optical fiber. **e** Photo and microscope view of the natural silk optical fiber. **f** Photographic images of the back and front sides of the device. **g** Demonstration of optical stimulation in a brain tissue phantom. **h** An image of a freely moving mouse that has been implanted with a flexible Silk-Optrode device

**Fig. 2:**
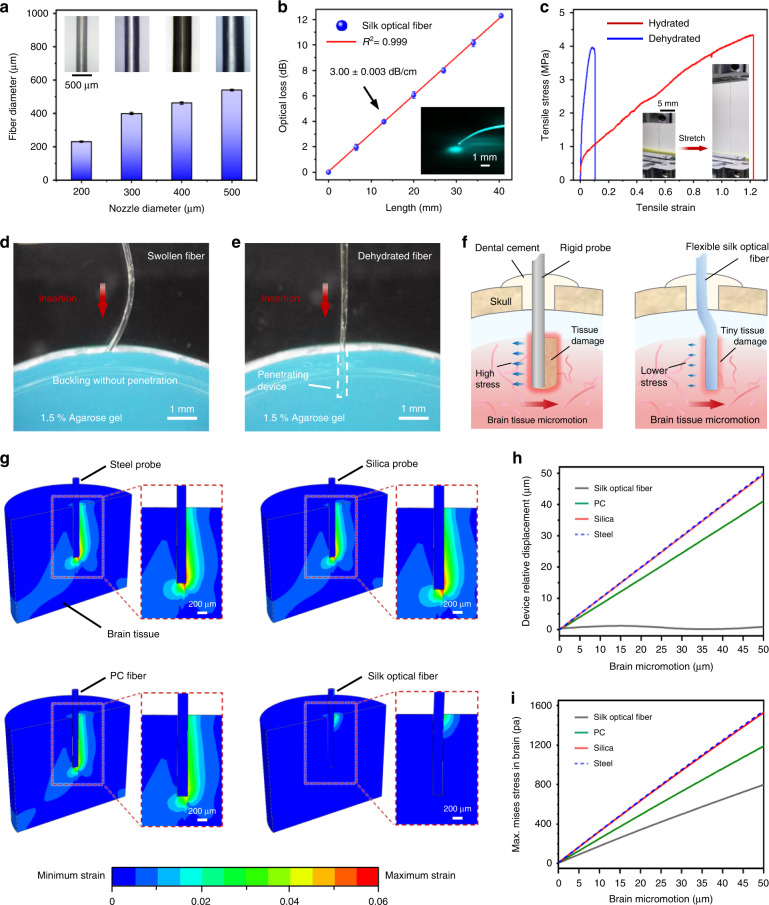
Characterization of the silk optical fiber in the flexible Silk-Optrode device. **a** The nozzle with different diameters corresponds to the diameter of the optical fiber. The error bars represent the standard deviation (*n* = 6). **b** Optical transmission of the optical fiber. The error bars represent the standard deviation (*n* = 3). **c** The strain‒stress curve of the silk optical fiber in the hydrated and dehydrated states. Inset, typical hydrated silk optical fiber before and after stretching. d) Insertion of the silk optical fiber in a fully wet (**d**) and dehydrated (**e**) state into the phantom brain (1.5% agarose) at a speed of 1 mm s^−1^. **f** A conceptual illustration of the probe implanted into brain tissue. Compared with the rigid probe, the hydrated flexible silk optical fiber can effectively reduce tissue damage and stress caused by micromotion between the brain tissue and nerve interface. **g** Maximum strain fields within the brain tissue during 50 µm lateral micromotion for implants of steel, silica, PC fibers, and flexible silk optical fibers. **h** The relative displacement for steel, silica, PC fibers and for the flexible silk optical fibers during 0–50 µm lateral micromotion of the brain tissue. **i** The maximum von Mises stress in the brain from steel, silica, PC fibers and flexible silk optical fibers during 0–50 µm lateral micromotion of the brain tissue

Natural silk has the advantages of high transparency and controllable mechanical strength and has gained new interest and breakthroughs in the field of biological optical devices^26^. Compared to materials commonly used for implanted optical fibers (for example, quartz and polycarbonate), silk offers the benefits of biocompatibility, adjustable mechanical properties, and easy processability into a variety of architectures (for example, films, fibers, gels, and blocks)^27,30,31^. To produce the Silk-Optrode probe (Fig. 1a–c), we first fabricated uniform silk optical fibers with a length of several tens of centimeters. Reliable production of highly transparent silk optical fibers is difficult because of the presence of air bubbles within the bulk materials^32,33^. In this work, we successfully manufactured a uniform high aspect ratio, high-transparency silk optical fiber (diameter 200–500 μm, length >200 mm) by preconcentrating the silk solution and carefully releasing meso- and microscale air bubbles in a controlled manner^34^ (Fig. 1d, e). As shown in Fig. 1d, the preconcentrated silk solution was first released into the methanol solution at a uniform speed through a custom-diameter nozzle. This step induces the secondary structure transition (from an alpha helix to a beta sheet^27^) within the silk and regulates the solubility to form the stable and insoluble fiber^34^. After molding, the product was transferred to deionized water for cleaning and soaking to remove residual chemicals. After these two treatments, the silk optical fiber shows excellent mechanical properties and high uniformity (Fig. 2a). We also conducted in vitro experiments with the fiber immersed in PBS solution for days and showed no apparent diameter change (Fig. S1), which proves its ability to maintain stable morphology. We test and calculate the propagation loss of the silk optical fiber through the cut-back method (in a wet state), yielding a competitive performance of 3.0 dB/cm in propagation losses (Fig. 2b) in comparison to polymer-based optical fibers previously reported^15^. This indicated that the silk optical fibers can deliver sufficient laser power for optogenetic stimulation.

Importantly, the formed silk optical fiber can be soaked in water to achieve the transition from a hard brittle state to a high elongation state. This phenomenon can be attributed to the destruction of hydrogen bonds in silk by water molecules^35–38^. The mechanical properties of wet and dehydrated silk optical fibers were tested through tensile-fracture experiments (Fig. 2c). The curve results show that the elastic modulus of the dehydrated fiber is 38.7 MPa, while the elastic modulus of the wet fiber decreases to 3.53 MPa. Owing to the change in Young’s modulus of the material, the bending stiffness of the 200 μm diameter silk optical fiber decreases from 3.05E-09 N·m^2^ to 2.77E-10 N·m^2^, which is 4 orders of magnitude lower than that of the quartz fiber with the same diameter (Fig. S2). At the same time, the ductility of wet fiber is greatly improved compared to dehydrated fiber. We therefore explored the application prospect of silk optical fiber in self-adaptive implantation into brain tissue: (a) before implantation, when the silk optical fiber is in the normal state of dehydration, the fiber has a high Young’s modulus to support itself to insert into the brain tissue; (b) after implantation, the silk optical fiber is transformed into a flexible and highly malleable state when exposed to cerebrospinal fluid, which self-adapts to brain tissue after implantation and reduces dislocation movement and damage (Fig. 2f). We further demonstrated this feature of silk optical fiber by simulating the implantation in vitro using the 1.5% agarose gel (Fig. 2d, e and Fig. S3). Experimental results show that fibers that are not hydrated can be implanted into the agarose gel without hindrance. With the increase in hydration time, fibers exhibited more obvious bending and softening during implantation. After 15 min of hydration, the fibers were sufficiently softened to be unable to implant into the agarose gel. The softening rate of the fiber is ideal, leaving a sufficient window for implantation while avoiding a prolonged rigid state of the probe and increasing tissue damage.

Herein, we quantitatively simulated the probe–tissue interactions after implantation using finite element analysis (FEA). Owing to the cylindrical geometry of the silk optical fiber probes and low adhesion between wet silk optical fibers and biological tissues, the mechanical properties of the aforementioned interactions after self-adaptive implantation are predominantly determined by the bending of the probes (with negligible effect from torsion). Accordingly, we developed a 3D FEA model simulating the experimental situations and physiological conditions of brain micromotion (see “Methods” section for details on FEA setups and simulating parameters). In this model, a silk optical fiber probe is inserted into a simplified brain model with a 2 mm implantation depth. Then, the backend of the probe is tethered to the skull, and the brain tissue moves along the lateral direction with 50 μm amplitudes, simulating probe bending and dislocation due to physiological brain micromotion after implantation. Contrastive numerical analysis with the same FEA model and experimental situation among the probes composed of silk, polycarbonate (PC), silica and steel were conducted. Figure 2g shows the strain fields of the whole 3D model when these different probes and fibers moved along the brain for 50 μm after 2 mm implantation. Notably, only the silk optical fiber demonstrates an agreeable mechanical match to the brain tissue after implantation, with strains in the brain substantially decreasing from the tethered backend to the probe tip and being negligible in most of the implanted section. Comparatively, strain fields in the other three situations stay relatively larger along the implantation direction and concentrate in the tip. Correspondingly, the relative displacement curve of the probe tip and brain shows the well-attached movement between the silk optical fiber probe and the brain and distinct dislocation in the other three situations (Fig. 2h). In addition, the maximum stress in the brain with silk optical fiber implantation remained relatively lower than that in the other three situations throughout the brain micromotion (Fig. 2i and Fig. S4). Attached movement and negligible stress fields observed for silk optical fiber probes in their self-adaptive implantation are anticipated to improve their biocompatibility and reliability.

### Fabrication and electrical characterization of the flexible electrode array

To integrate high channel count electrodes on the surface of the Silk-Optrode probe with a negligible increase in the total device volume, we designed and fabricated an ultra-flexible electrode array using microfabrication techniques (Fig. 1a, b), as previously described^13^. Each flexible electrode array consisted of 128-channel 100-nm thick gold microelectrodes that were evenly distributed along four polyimide (PI) shanks with 2.5-μm-thickness, 105-μm- width on average, and 5-mm- length (Fig. S5). The bending stiffness of the electrode array is ~4.23 × 10^−13 ^N·m^2^, approximately five orders of magnitude lower than that of commonly used silicon probes ^7^, and three orders of magnitude lower than that of silk optical fibers (Fig. S2). Meanwhile, the volume of the implanted part of the microelectrode array is only 2.713 × 10^5^ μm^3^, which is approximately two orders of magnitude lower than that of the silk optical fiber (3.14 × 10^7^ μm^3^). The electrode array is thin and flexible enough to be easily bent and attached to the curved surface of the fiber without notably increasing the probe diameter (Fig. 1b, c). Microelectrodes on the electrode array were electrically addressable through substrate supported bonding pads, and the electrical connection between the array and measurement electronics was made by flip-chip-bonded flexible printed circuits. After the flexible electrode array is bonded to the electronic interface and released by nickel etchant, we use a prefabricated mold to align it with the center axis of the optical fiber. The Silk-Optrode probe was assembled under a microscope. We used a polyethylene glycol (PEG, molecular weight of 30,000) solution as a biological binder to fix the four shanks of the flexible electrode array radially to the quadrupole of the silk optical fiber and align the tips. A silk protein solution can also be used as the bio-glue. The Silk-Optrode device was then held in a position for drying in air to enhance the bonding between the electrode array and the hosting probe. Before neural recording, the conductive polymer polyethylenedioxythiophene (PEDOT) was electrodeposited on the recording sites (Fig. S6a–e). The nanoscale roughness of the PEDOT films effectively increased the active surface areas of the microelectrodes (Fig. S6d), which is consistent with previous research^39,40^. As a result, the interfacing impedance between the microelectrodes and the surrounding liquid was decreased by a factor of ~10 (at 1 kHz), which can effectively reduce the thermal noise during neural recording^41,42^.

### Simultaneous optogenetic stimulation and electrical recording of neuronal activity

**Fig. 3:**
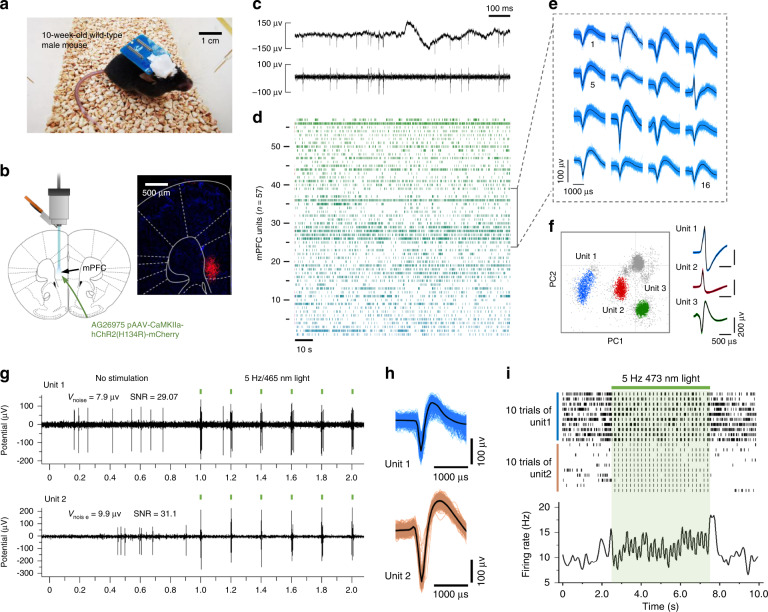
Demonstration of simultaneous modulation and recording of neural activities. **a** A photograph of a mouse implanted with the silk optical fiber-based Silk-Optrode probe. **b** Optical stimulation and electrical recording in the mPFC of mice with a Silk-Optrode probe and expression of ChR2 in the mPFC for a mouse a month after viral injection. **c** Example raw data traces show local field potentials and spiking (above) and high-pass filtered signals (below). **d** Example spiking raster from a Silk-Optrode probe implanted in a single mouse. **e** Example spike waveforms from **d**. **f** Action potential amplitudes on one single channel from a Silk-Optrode probe demonstrate three amplitude clusters (color coded). Average action potential waveforms shown next to the PCA. **g** Light stimulation of the mPFC projection evoked action potentials of the units in **h** and a complex multiunit response. **h** Average action potential shape is shown for the recording in **g**. **i** Raster of the unit activities in **g** and mean firing rate versus time of a cortical neuron from a ChR2-expressing mouse during application of 5 s-Hz light pulses

We tested the capability of our flexible Silk-Optrode probe to integrate optical stimulation and multichannel electrical recording of neuronal activity in mouse brains. Mice were injected with AG26975 pAAV-CaMKIIa-hChR2(H134R)-mCherry in the medial prefrontal cortex (mPFC) of the left hemisphere and were housed for at least 4 weeks for the virus to be expressed (Fig. 3a, b). The mice were then implanted with a Silk-Optrode probe, and their neural activities were recorded weekly. We obtained high-quality multichannel recordings with the flexible Silk-Optrode probe in awake, free-moving mice (Fig. 3c and Fig. S7). A mouse with 109 channels (85% fabrication yield) implanted in the mPFC isolated the activity of 57 putative individual units during the OFT of 10 min after 1 week of implantation, which yielded up to 0.52 units per channel (Fig. 3d, e), considerably higher than similar types of neural probes^43–45^. Unit signal sorting was carried out through principal component analysis (PCA), and three typical well-separated neuronal units are shown in Fig. 3f, demonstrating the ability of a single channel to record high-quality signals from multiple neurons.

In systems neuroscience and clinical medicine, intracranial stimulation is usually achieved by conventional electrodes, but it has proven difficult to determine the local nerve response to stimulation due to the electrical artifacts caused by electrical stimulation, especially at higher frequencies. In contrast, optogenetic stimulation allows us to avoid electrical artifacts while recording local neural activity. We explored the integration of electrical recording with optical stimulation enabled by our Silk-Optrode devices in awake behaving mice. These mice injected in the mPFC with AAV containing a ChR2 fusion protein were subjected to an 18-min open-field test (OFT), during which 180-s epochs of optical control were separated by 180-s intervals of no light delivery. The epochs of optical control consisted of pulsed laser light at various frequencies (laser wavelength *λ* = 465 nm, pulse width 2 ms, power density 20 W mm^−2^). Typical optical stimulation-induced neural activities were observed with intervals of 200 ms, matching the frequency of laser pulses (5 Hz). Notably, the single units remained well-isolated during stimulation, reflecting the retention of signal recording quality during stimulation (Fig. 3g–i). In addition to the single unit induced by the laser pulses, the stimulation produced complex multiunit responses that preceded (but no interference) the synchronous emission of units (Fig. S8) observed in free-moving mice, consistent with previous studies^5,29^. The same experiment of simultaneous optogenetic stimulation and electrical recording was performed on control mice transduced with AG26975 pAAV-CaMKIIa-mCherry, and no significant additional light-caused noise was found before and during optical stimulation (Fig. S10). The above results prove that the flexible Silk-Optrode probe can achieve reliable optogenetic modulation of electrically recorded neurons, a feature highly dependable for the analytics of neural circuits.

### Behavior studies during optical stimulation

**Fig. 4:**
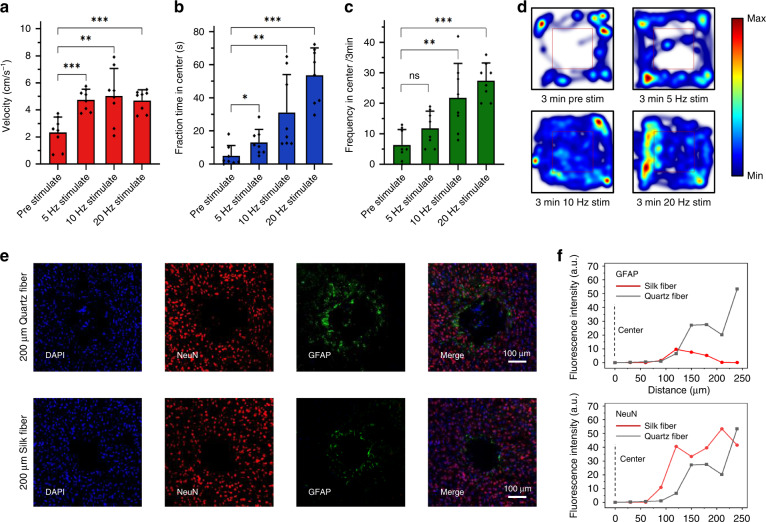
Behavioral effects of silk optical fiber-based Silk-Optrode probe stimulation during the OFT and device implantation impact the brain. **a**–**c** Velocity, fraction time, and frequency in the center/3 min of AG26975 pAAV-CaMKIIa-hChR2(H134R)-mCherry mice in the mPFC with or without optical stimulation (32 OFTs). The error bars represent the standard deviation. **d** Representative heatmap images tracing the position of mice transfected with ChR2. The central area is marked with a red box. **e** Immunohistochemistry study of different implantable devices. Confocal fluorescence images of 20 µm-thick slices for astrocytes (GFAP, green), neurons (NeuN, red), and nuclei (DAPI, blue). **f** The fluorescence intensity of GFAP and NeuN was plotted against the distance from the implant center of neural probes using the silk optical fiber and quartz probe, respectively

The Silk-Optrode device is light enough (with a weight that is less than 2.2 g) to avoid interfering with the movement of mice under freely behaving conditions. Experiments to demonstrate the capability of the device to conduct behavior regulation on freely moving mice (transduced with AG26975 pAAV-CaMKIIa-hChR2(H134R)-mCherry or a control virus AG26975 pAAV-CaMKIIa-mCherry in the mPFC) were then performed through OFT. We analyzed three aspects of the behavior during the 32 OFT experiments: mice velocity, time of the mice being in the central area, and frequency of the mice entering the central area (Fig. 4a–c). We calculated the average value of the three indicators prior to the first optical stimulation epoch, as well as during different frequency stimulation epochs, for each OFT. Compared with the condition without stimulation, the three indices were improved to different degrees when the ChR2-expressing mice were subjected to 5, 10, and 20 Hz laser stimulation. The speed of mice under light stimulation was obviously increased compared with that before stimulation and tended to be flat with the change in stimulation frequencies. The time spent in the central area and the frequency of entering the center increased with increasing stimulation frequencies in the range of 5–20 Hz. These results matched the activity heatmaps and trajectories of mice during the OFT (Fig. 4d and Fig. S9), showing a positive contribution of stimulation to anxiety resistance of mice expressing ChR2 in excitatory neurons, consistent with previous studies^46–48^. The same behavioral experiment was performed on control mice transduced with AG26975 pAAV-CaMKIIa-mCherry, and no significant differences were found before and after stimulation (Fig. S10). The preliminary results of behavior studies suggest great potential to applying the Silk-Optrode devices on freely moving animals.

### Assessment of chronic stability and biocompatibility

The biocompatibility of the Silk-Optrode devices and their chronic stability were investigated through standard immunohistochemical staining of neurons. To evaluate the invasiveness and glial responses, neural probes made of a flexible silk optical fiber (200 µm in diameter) and a commercial optical fiber (quartz material, 200 µm in diameter) were implanted into the brains of mice. Immunohistochemical analysis was performed by quantifying the glial response 2 months after surgery (Fig. 4e). Compared with commercial quartz fibers, Silk-Optrode probes produced less glial scarring and microglial aggregation, as well as less neuronal apoptosis (Fig. 4f and Fig. S11). This can be attributed to the mechanical compliance of the flexible silk optical fiber and small overall tissue displacement reducing injury and the immunoreactive glial response induced by brain inserts^45,49,50^. The device shows great potential for adaptive implantation, which reduces the difficulty of implantation, chronic tissue lesions and immunoreactivity. Continuous observation of potentials correlated with laser pulses after operation also proved the chronic stability of the device (Fig. S12).

## Discussion and conclusion

In this study, we have developed a flexible multifunctional system based on a natural biological material, silk, for optogenetic manipulation and the simultaneous recording of multichannel electrophysiological signals from the brain. A typical Silk-Optrode probe consists of a flexible natural silk optical fiber and ultrathin microelectrode arrays. The probe achieved the integration of 128 recording channels in the space of 200 microns in diameter and 2 mm in length, while Young’s modulus was only ~3.53 MPa. The microelectrode arrays were manufactured through microfabrication processes, with 128 recording channels, a thickness of only 2.5 microns and a bending stiffness of only 4.23 × 10^−13^ N·m^2^. Meanwhile, we manufactured a high aspect ratio (diameter 200–500 μm, length >200 mm), high-transparency (propagation loss of 3.0 db/cm) silk optical fiber through an improved process. We found that the silk optical fiber can be immersed in water to dramatically reduce its Young’s modulus and bending stiffness (~11 times), transforming itself from a hard and brittle state to a highly malleable state. The wet fiber is highly flexible, with a Young’s modulus of only ~3.53 MPa. Based on this phenomenon, we concluded that silk optical fibers can be inserted into brain tissue by their stiffness under dehydration. After contact with tissue fluid, the silk optical fibers become soft and match the mechanical properties of the brain tissue. We further demonstrated this by simulating the implantation environment using the AGAR gel. The results of finite element analysis showed that compared with the solid polymer, glass or metal probe of the same size, the silk optical fiber effectively reduced the relative micromotion and tissue damage to the brain. This integration of biocompatibility, good tissue-device interaction, and self-adaptive properties may provide a promising direction for the development of other multifunctional brain computer interface devices.

Owing to the mechanical properties of the device matching brain tissue and the reduction of impedance resulting from electrochemical modification, the Silk-Optrode probes performed well during in vivo recording. Simultaneous optogenetic stimulation and electrical recording of neuronal activities demonstrate that silk optical fiber-based Silk-Optrode probes can be a promising tool in the field of behavior and neural circuits^44,51^. In the next steps of this work, we will try to use cellulose nanofibers mixed with natural silk as an optical waveguide material to obtain a lower optical transmission loss^52^. Based on the easy structural processing of silk materials, we can further improve the functionality of Silk-Optrode probes: the surface of silk fibers can be combined with biomimetic micro-terrain structures to decrease the immunoreactive glial response^53,54^. Sharpening the tip of the fiber makes it possible to implant the probe without cutting through the dura, which is beneficial in reducing the risk of intraoperative injury and infection^55,56^. In addition, recent studies have demonstrated that natural silk can be used as an excellent biological function carrier^27^. A wide variety of functional viruses^57,58^ and drug molecules^14^ could be embedded into the silk carrier of our Silk-Optrode system. Highly localized drug delivery around the device-tissue interface will be the target of our next exploration. The Silk-Optrode probes also have the potential to sense other types of biological signals in addition to extracellular potential signals. For example, by coating protective films containing enzymes on the surface of microelectrodes, the device can achieve the sensing of neurotransmitters such as glutamate. By replacing part of the gold recording sites of the microelectrode array with platinum stimulation sites, the probe will have the potential for both intracranial signal recording and stimulation^59^. At the same time, we can try to optimize the morphology of the electrode array to make the electrode sites more evenly distributed in the space around the end of the fiber and obtain a higher unit yield. For instance, multibranched networks with a self-unfolding potential can be obtained^12,60^. These developments will provide valuable guidance for high-precision neural circuit analysis and multifunctional biosensing platforms in neuroscience.

## Experimental section

### Preparation of lyophilized silk fibroin

Lyophilized silk fibroin was prepared using previous purification methods^34^. Chopped *B. mori* cocoons (10 g) were degummed by boiling them in a 0.02 M Na_2_CO_3_ (Sigma-Aldrich, USA) solution (4 L) for 60 min. The degummed silk was rinsed in deionized water for 3 × 20 min and dried for more than 12 h. Then, the dry silk (10 g) was dissolved in a 9.3 M LiBr (Sigma-Aldrich, USA) solution (40 mL) at 60 °C for 4 h. Afterward, the solution was dialyzed (Pierce, USA) in deionized water for 48 h and centrifuged for 2 × 20 min at 12,000 r.p.m to obtain the cleaned silk solution in water. Then, the silk solution was frozen at −80 °C for 12 h and freeze-dried until the water completely sublimated.

### Fabrication of the silk optical fibers

The purified lyophilized silk proteins were then dissolved with HFIP (Sigma-Aldrich, USA) at initial concentrations of 23% (w/v). The solution was shaken at 37 °C for 12 h until the silk proteins were entirely dissolved. The silk protein solution needs to be placed in a fume hood for 48 h to vaporize the HFIP. By this method, the concentration of the silk protein solution can be increased by ~1.5 times. The solution was allowed to settle for 12 h to release the bubbles and then transferred to a 10 mL syringe. With the help of a microinjector (Harvard Apparatus, USA), the solution was released uniformly into the methanol solution through a predetermined nozzle at a rate of 10 μL/s to solidify and form the optical fiber. After solidification, the silk optical fiber was soaked in methanol for 3 days to achieve maximum structural transformation. The silk optical fiber was placed in deionized water for another 7 days to replace the residual methanol with water.

### Characterization of the silk optical fiber

The surface of the silk optical fiber was observed, and its diameter was measured through optical microscopy (Olympus, Japan). The optical loss of an optical fiber in air is measured through the cut-back method. The fiber samples were measured in length and immersed in deionized water for 20 min to fully moisten. The silk optical fiber is then mounted on a prefabricated base and coupled to the laser generator (RWD, China) via an optical waveguide. The other end of the fiber is connected to an optical power meter (Thorlabs, USA) to measure the light output, and the angle of the fiber tip is fixed through a mold. The measurement range of the fiber length is between 0 and 4.2 cm (0.7 cm interval). The strain‒stress curves of the silk optical fiber in the wet and dehydrated states were measured by a CMT4204 test machine (SUST, China) in the tensile breakage test.

### FEA analysis of brain micromotion

Finite element analysis was used in the commercial software ABAQUS (ABAQUS Analysis User’s Manual 2020). The brain tissue was represented by incompressible hyper-elastic neo-Hookean material with an initial shear modulus of 5.5 kPa and modeled with an 8-node, hybrid, reduced integration 3D brick element (C3D8RH)^61^. Probes composed of silk optical fibers and other materials were modeled with an 8-node 3D brick element (C3D8) with corresponding mechanical properties. Specifically, the elastic modulus (E) and Poisson’s ratio (ν) are ESilk optical fiber = 3.53 MPa, νSilk optical fiber = 0.49; EPC = 2.38 GPa, νPC = 0.37; ESilica=50 GPa, νSilica = 0.17; ESteel=193 GPa, νSteel = 0.265. The mesh convergence of the simulation was guaranteed for all 3D models. Before micromotion, the top surface of the implanted probe was fixed, while another end was open for deflection. To simulate brain micromotion, the bottom surface of the brain tissue was loaded with a 50 µm lateral displacement in a general static procedure step. The interaction configuration between the probe and the brain tissue was set to be surface-to-surface contact as tangential friction with a friction coefficient of 0.3^62^.

### Fabrication of microelectrode arrays

Engineering flexible microelectrode arrays were fabricated using standard microfabrication processes. The key fabrication steps are as follows: a layer of 2 μm thick silicon dioxide was first thermally grown on a silicon wafer (n-type 0.005 V·cm, XiaMen LuYuan Science and Technology, China), and then a 100-nm-thick nickel sacrificial layer was patterned through photolithography and E-beam evaporation. A 1.2-μm-thick layer of PI (PI-2610, HD Microsystems, USA) was spin-coated on the wafer and cured at 380 °C for 12 h in a nitrogen oven as the bottom and top insulating layers. The interconnects, microelectrodes, and bonding pads were fabricated through photolithography and metal deposition with a 5-nm-thick chromium layer, a 150-nm-thick nickel layer, and a 50-nm-thick gold layer. With aluminum hard mask protection, the wafers were then subjected to an RIE etching process to expose the microelectrode points and bonding pads. The wafer was etched by an aluminum etchant to remove the hard mask protection layer. The bonding pads of the 128-channel devices were flip-chip bonded to custom 0.8-mm-thick PCBs (Shenzhen JDB Electronic Technology, China) through reflow welding, and then the nickel etchant was used to release the flexible microelectrode arrays from the silicon substrate. The structural design of the microelectrode array is similar to that of the electrode array in a previous work^11,63^. To facilitate bonding to the PCB, the tail end of the flexible microelectrode array is retained on the wafer substrate instead of being completely separated from it. Each electrode recording point of the microelectrode array was electrically connected to the bonding pad on the wafer substrate. We can address and record 128 electrode channels simultaneously through two 64-pin Molex interfaces on the PCB.

### Electrochemical deposition of PEDOT

The electrodeposition of PEDOT on the surface of the exposed electrode was performed at an electrochemical workstation (CH Instruments, China). The solution of 0.01 M ethylenedioxythiophene (EDOT, Sigma-Aldrich, USA) and 0.1 M sodium dodecyl benzene sulfonate (Sigma-Aldrich, USA) was prepared in a 20/80 vol% mixture of isopropyl alcohol and water. The microelectrode arrays were connected to the working electrode sites. The reference electrode (Ag/AgCl electrode) and counter electrode were connected to a platinum wire in contact with the solution. The deposition was carried out in potentiostatic mode at 0.8 V, at room temperature. Then, the microelectrode arrays were soaked in deionized water for 24 h to remove excess electrolyte and subsequently dried for use.

### Assembly of the Silk-Optrode probe

The silk optical fibers were placed in a fume hood for 3 h to be completely dehydrated. The fiber was then cut to 6 mm length and inserted into a 500 μm diameter through hole in a prefabricated mold, and the gap between the fiber and the hole was filled with 5 min epoxy. The nonimplanted end of the fiber was embedded in a ceramic sleeve for coupling to the optical waveguide of the laser generator. The silicon substrate of the electrode array was held in place by a prefabricated snap on the mold and aligned with the center of the fiber. Under the microscope, a PEG (30,000 molecular weight, Sigma-Aldrich, USA) solution was used as a biological binder to fix the four shanks of the flexible electrode array radially to the quadrupole of the silk optical fiber and align the tips. Tungsten wires with a diameter of 50 microns are used as tools to fine-tune the position of the electrode array during assembly. The device was then placed in a fume hood and dried for 3 h.

### Implantation of the Silk-Optrode probe into mouse brain

All animal procedures were performed at Shanghai Laboratory Animal Research Center, Shanghai, China. All animal tests were approved by the Institutional Animal Care and Use Committee of Fudan University (approval number: 2019-07-HSYY-SZF-01). Male adult C57BL/6 mice were used for the current study. The virus (AG26975 pAAV-CaMKIIa-hChR2(H134R)-mCherry, HeYuan, China) was delivered into the mPFC (1.95 AP, 0.3 ML, −2.2 DV), followed by the implantation of the Silk-Optrode probe after 1 month. The animals were placed in a standard rodent stereotaxic frame, positioned using ear bars, and anesthetized using isoflurane (3% for induction and maintained at 1 to 2%) in medical-grade oxygen. The skull was exposed and prepared by scalping the crown and removing the fascia. A round craniotomy of 500 μm in diameter was performed above where the virus was injected, and the dura was carefully removed. The Silk-Optrode probe was fixed in a custom holder that was mounted on a micromanipulator. The microelectrode array was then implanted into the mPFC of the mouse at a speed of 0.5 mm/s and a depth of 2.2 mm. The device was then fixed to the skull with dental cement. The mice were allowed to recover for at least one week.

### Simultaneous optogenetic stimulation and electrical recording of neuronal activity

Laser stimulation (465 nm, 2 ms pulse width, 20 mW) was performed by an optogenetic stimulation system (RWD, China). Voltage signals from the neural electrodes were amplified and digitized using the RHD Recording System (Intan Technologies, USA) with a 200 μm diameter stainless steel wire inserted into the contralateral hemisphere of the brain as the grounding reference. The neural signals are transmitted through data lines, and the head stage of the recording system interconnects with the PCB of the device through two 64-pin Molex interfaces (Molex, USA). The sampling rate was 30 kHz, and a 300-Hz high-pass filter and a 50-Hz notch filter were applied for single-unit recording. Spike detection and spike sorting were performed using an Offline Sorter (Plexon, USA).

### Open-field tests

OFTs were carried out with C57BL/6 mice injected with AG26975 pAAV-CaMKIIa-hChR2(H134R)-mCherry in the mPFC. All trials (18-min session, 3-min OFF/ON/OFF epochs, 2-ms pulse width) were recorded by a camera. Video tracking software was used to analyze movement (SMART, Panlab, Spain).

### Immunohistochemistry and imaging

Two months after device implantation, the mice were administered an intraperitoneal injection of sodium pentobarbital and then transcardially perfused with 1× PBS and 4% paraformaldehyde (Sigma‒Aldrich, USA). The mice were decapitated, and the brain was carefully removed. The brain was cryoprotected in a 4% paraformaldehyde solution overnight and then sectioned into 20-μm-thick slices perpendicular to the probes. Brain slices were incubated in 0.3% Triton X-100 at 25 °C for 15 min to increase the permeability of the membrane. Then, the slices were incubated in a blocking solution consisting of 3% bovine serum albumin for 1 h at 25 °C. Afterward, the slices were incubated in primary antibodies (1:1000 dilution, 26975-1-AP + for neun, SanYin, China) overnight at 4 °C. After antibody incubation, the slices were rinsed three times with PBS. Then, the slices were incubated in HRP antibodies (1:200, goat polyclonal, KPL, USA) for 50 min. A TSA agent (1:500 dilution, CY3-HRP-tsa, ZhiShan, China) was dropped onto the slices and incubated for 10 min, after which the slices were rinsed with PBS. The secondary antibodies (1:100 dilution, BA0056 for gfap, Boster, China; 1:100 dilution, goat polyclonal anti-488, Jackson, USA) were incubated with the slices overnight at 4 °C, and nuclei were counterstained with 40,60-diamidino-2-phenylindole (DAPI, ZhiShan, China). Images were obtained and analyzed using fluorescence microscopy (Olympus, Japan).

## Supplementary information

Supplementary information
