# Supplementary material for: A silk-based self-adaptive flexible opto-electro neural probe
Source: Microsyst Nanoeng. 2022 Nov 8;8:118. doi: 10.1038/s41378-022-00461-4 (PMC9643444; doi:10.1038/s41378-022-00461-4)
Supplement: Supplementary file 1 — Supplementary information [file 41378_2022_461_MOESM1_ESM.docx]

Supporting Information

**A silk-based self-adaptive flexible opto-electro neural probe**

*Yu Zhou, Chi Gu, Jizhi Liang, Bohan Zhang, Huiran Yang, Zhitao Zhou, Meng Li, Liuyang Sun, Tiger H. Tao*, and Xiaoling Wei**


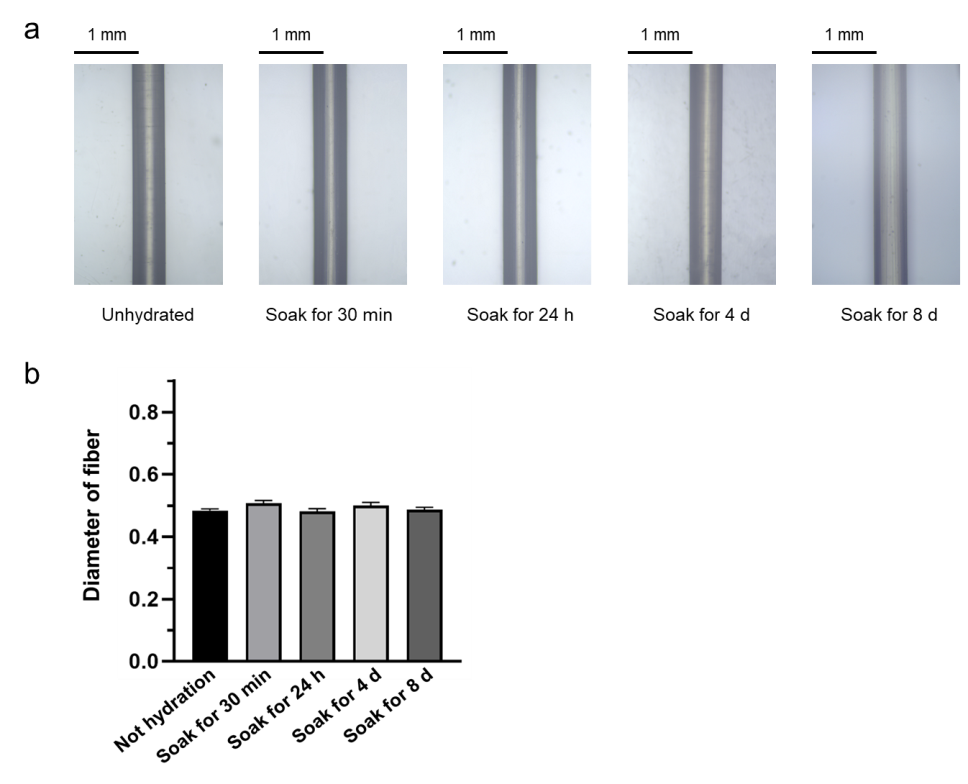


**Figure S1**. Diameter comparison of silk fibers in different states. (a) Microscopic photos and (b) measurement data.


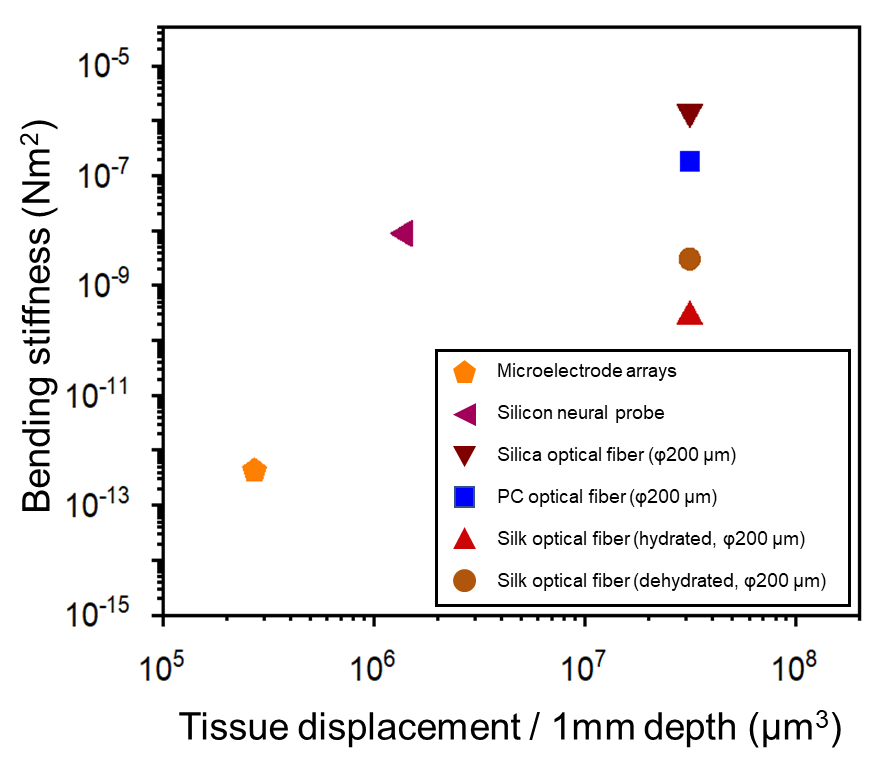


**Figure S2.** Drastically reduced bending stiffness and tissue displacement of the microelectrode arrays and Silk optical fiber compared with other representative probes. For shaft-shaped probes, K_s_=E_s_wh^3^/12, where E_s_ is the Young’s modulus of the shaft material and h and w are the thickness and the width of the shaft, respectively. For cylindrical probes, K_w_=E_w_πd^4^/64, where E_w_ is the Young’s modulus of the wire/fiber material and d is the diameter of the wire. TD/1 mm equals the average cross-section area multiplied by the implanted length of 1 mm.


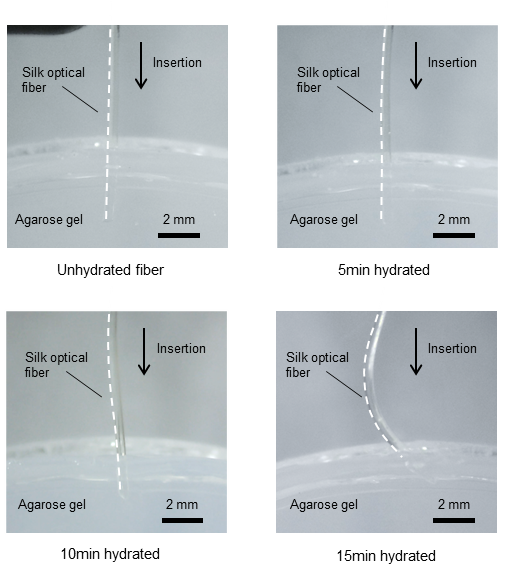


**Figure S3**. *In vitro* implantation test of silk optical fibers under different hydration times.


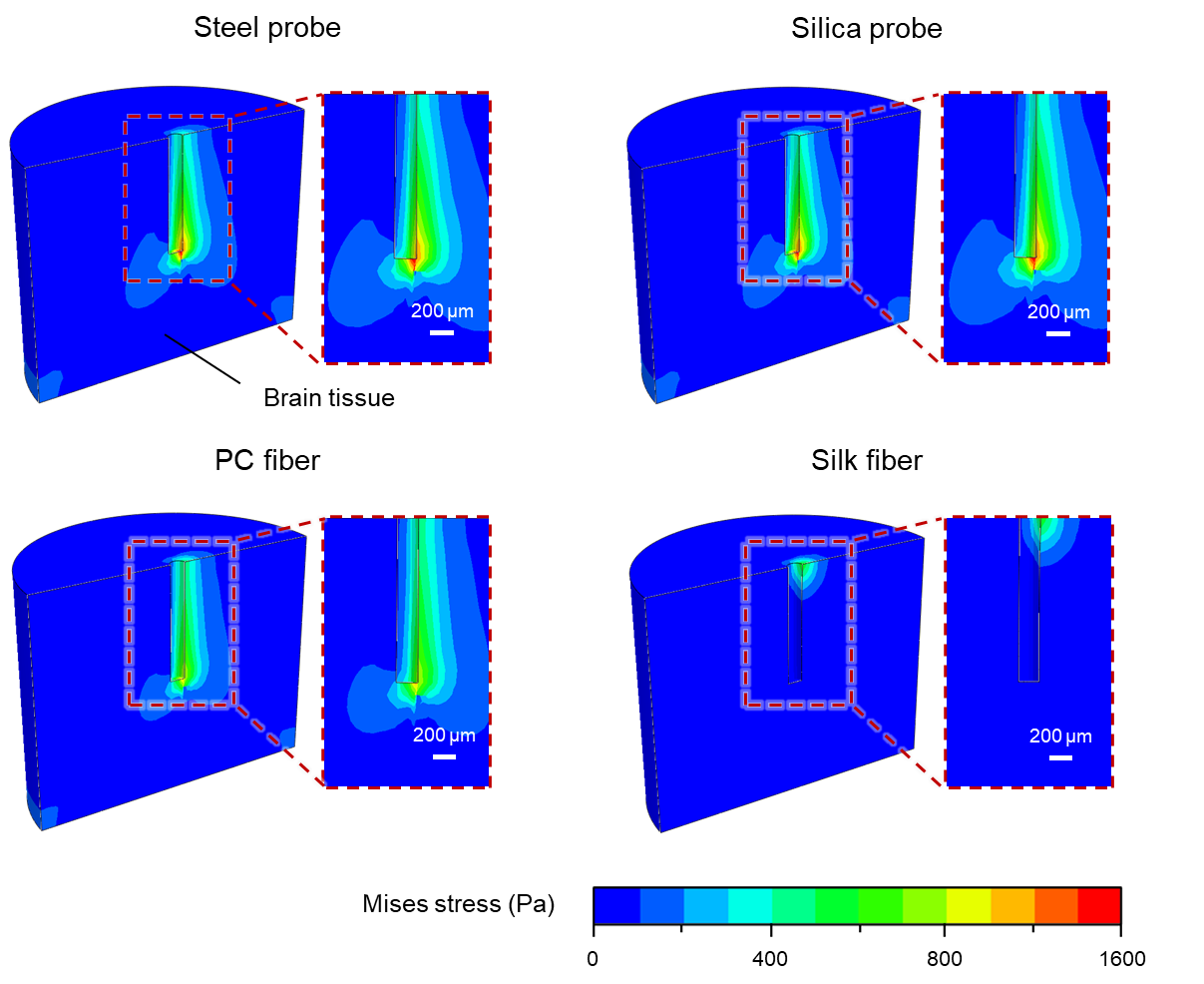


**Figure S4.** Mises stress profiles within the brain tissue during 50 µm lateral micromotion, for implants of steel, silica, PC fibers, and flexible silk optical fibers.


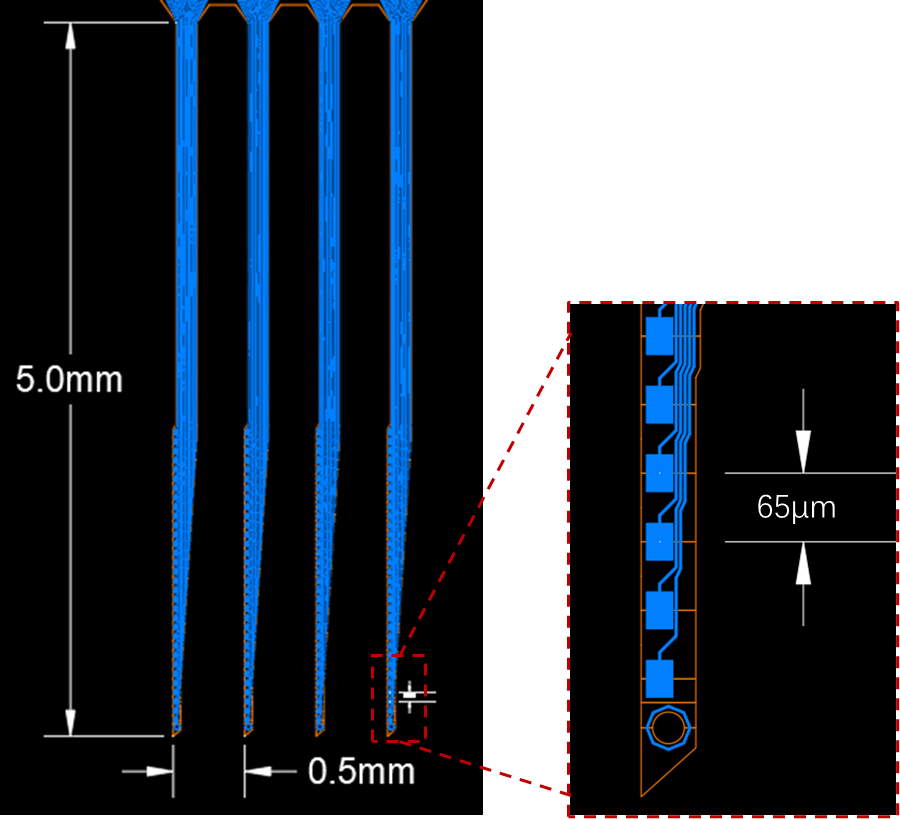


**Figure S5.** Device design of the electrode array. The microelectrodes are 35 × 25-μm in size.


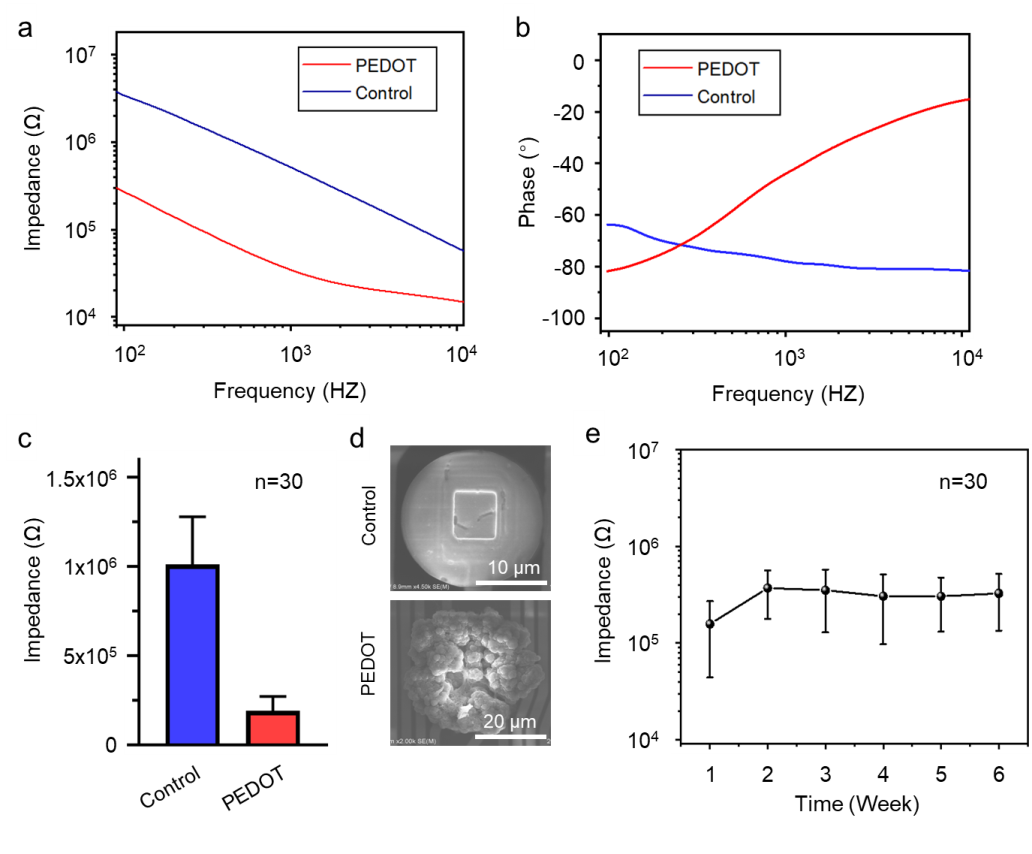


**Figure S6.** (a, b) Impedance and phase variations with frequency from a microelectrode before and after PEDOT modification. (c) Impedance statistics of microelectrodes before and after PEDOT modification. (d) SEM images of a typical gold microelectrode before and after PEDOT modification. (e) Impedance changes of microelectrodes during the six weeks after implantation.


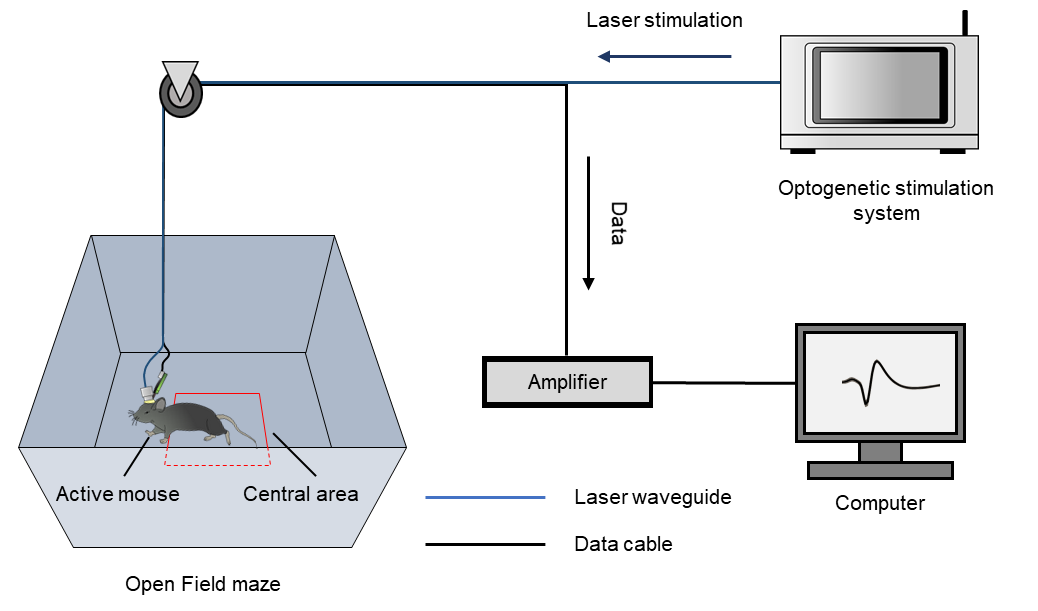


**Figure S7.** Simultaneous optogenetic stimulation and electrical recording of neuronal activity.


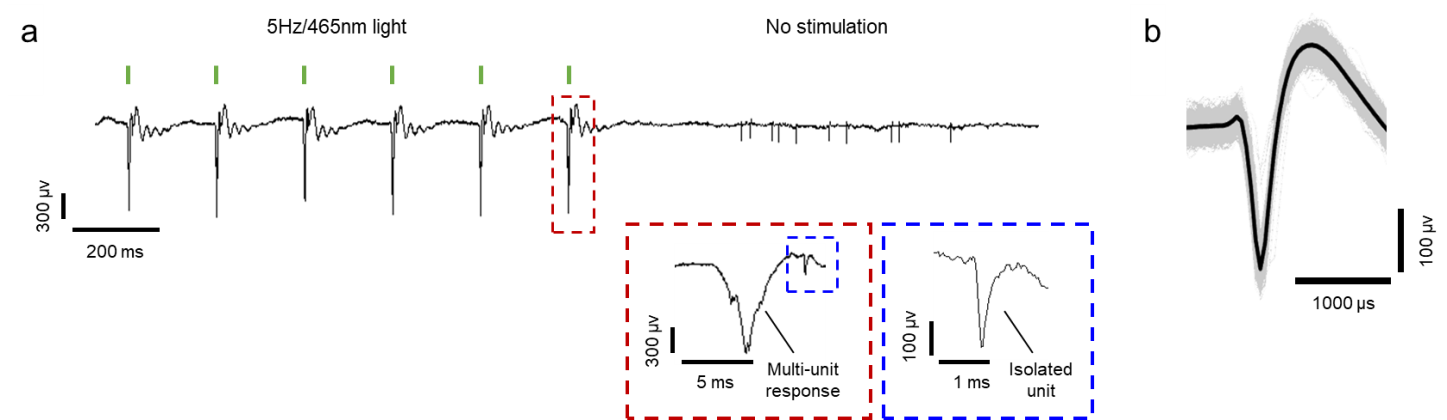


**Figure S8.** (a) Electrophysiological recording in the mPFC during 5 Hz electrical stimulation with a pulse width of 2 ms. (b) Sorted shapes of the action potential of the unit.


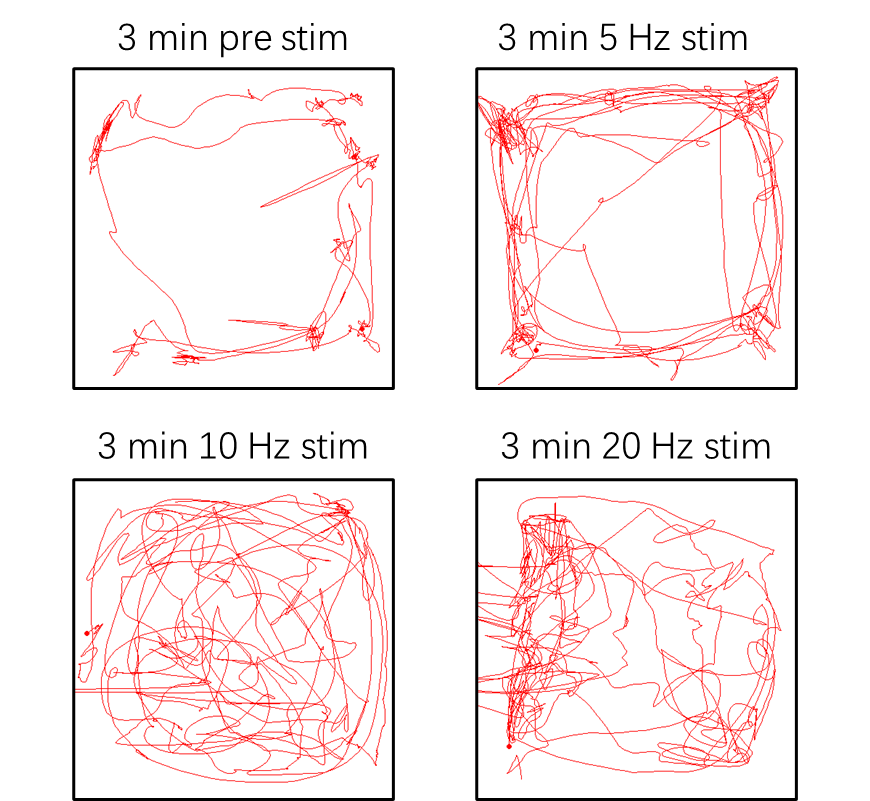


**Figure S9.** Representative traces of movement from the same mouse in the OFT without and with optical stimulation.


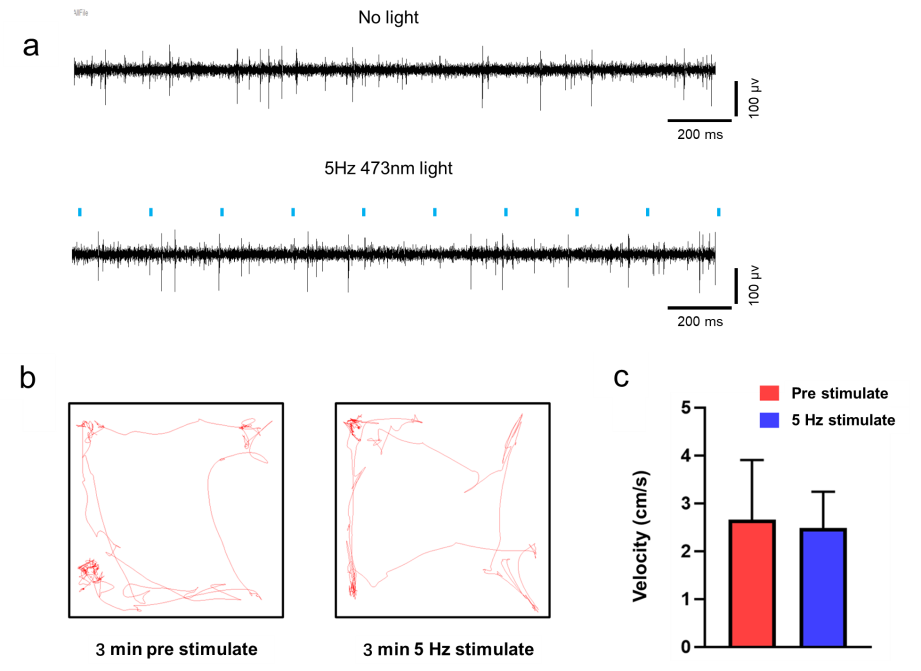


**Figure S10.** a) Simultaneous optogenetic stimulation and electrical recording in control mice transduced with AG26975 pAAV-CaMKIIa-mCherry (control virus without ChR2). (b) Representative traces of an AG26975 pAAV-CaMKIIa-mCherry mouse before and after 5 Hz stimulation. (a) The average velocity of AG26975 pAAV-CaMKIIa-mCherry mice before and after 5 Hz stimulation. No significant difference was found. n = 8, error bars represent the standard deviation.


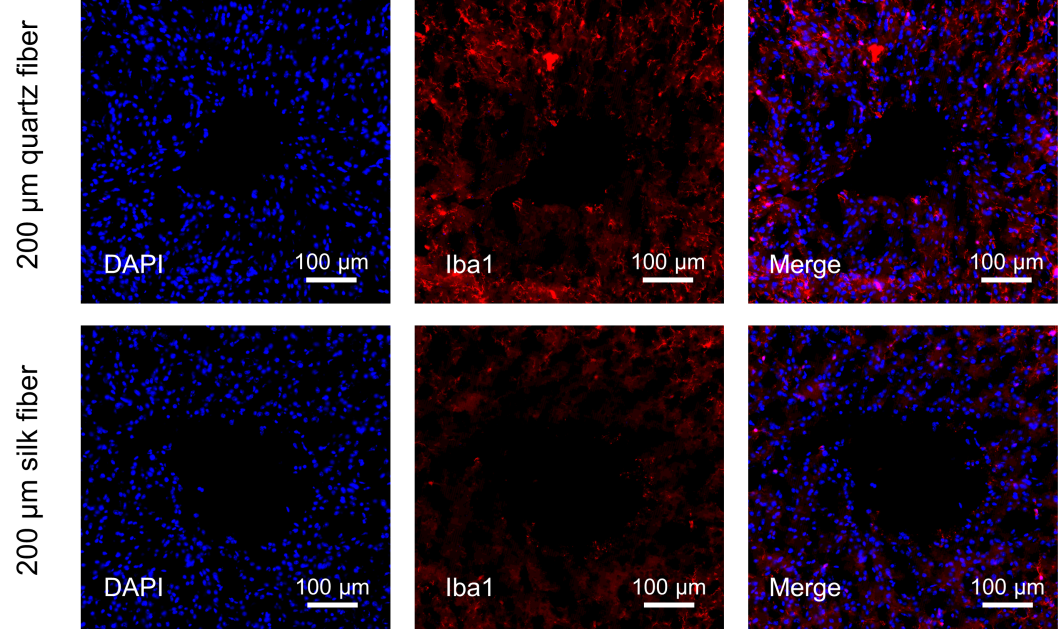


**Figure S11.** Immunohistochemistry study of different implantable devices. Confocal fluorescence images of 20 µm-thick slices for microglia (Iba1, red) and nuclei (DAPI, blue).


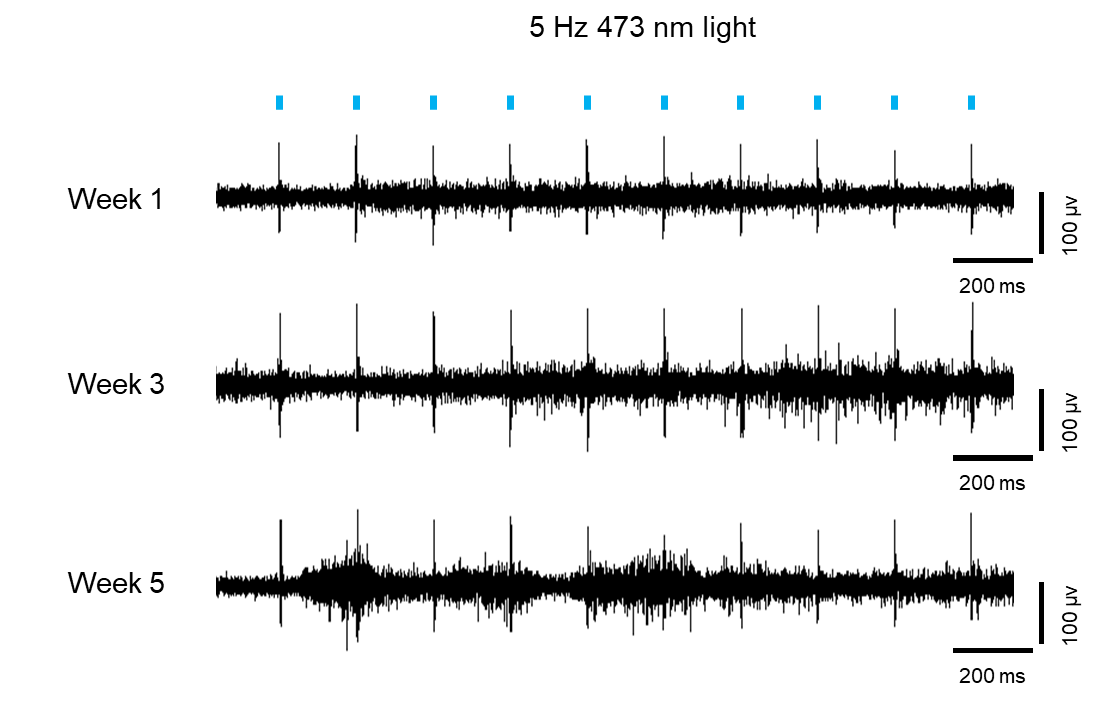


**Figure S12.** Representative potentials correlated with laser pulses observed 1–5 weeks after device implantation.
